# Challenges in the diagnostics and treatment of ectopic ameloblastic carcinoma: a case report

**DOI:** 10.3325/cmj.2020.61.271

**Published:** 2020-06

**Authors:** Marko Tarle, Danko Müller, Antonia Tarle, Igor Blivajs, Naranđa Aljinović Ratković, Predrag Knežević

**Affiliations:** 1 Department of Oral and Maxillofacial Surgery, University Hospital Dubrava, Zagreb, Croatia; 2 University of Zagreb School of Medicine, Zagreb, Croatia; 3 Department of Pathology and Cytology, University Hospital Dubrava, Zagreb, Croatia; 4 University of Zagreb School of Dental Medicine, Zagreb, Croatia

## Abstract

Ameloblastic carcinoma (AC) is a rare and aggressive malignant epithelial odontogenic tumor, most commonly located in the mandible or maxilla. An extremely rare extragnathic localization of AC with no connection to the jaws, ectopic ameloblastic carcinoma (EAC), has so far been described only three times. This report presents a 64-year-old male with skull base ameloblastic carcinoma and offers a review of diagnostic and treatment challenges related to EAC. Because of its rarity and histological similarity to other tumors, EAC is often misdiagnosed. This is why we established a pathohistological and immunohistochemical profile of EAC that differentiates it from histologically similar tumors. The most frequently used EAC treatment is radical surgical resection, but the majority of reviewed reports described local recurrence. Taking into consideration new scientific discoveries on the molecular pathogenesis of ameloblastoma, we are the first to have performed BRAF mutation analysis in an EAC patient. BRAF inhibitors offer promising results in the treatment of BRAF-positive ameloblastomas and should continue to be researched in AC and EAC patients. Finally, EAC should be considered in differential diagnosis of head and neck tumors outside the jaws.

Ameloblastic carcinoma (AC) is an exceptionally rare and aggressive malignant variant of ameloblastoma – a benign odontogenic tumor arising from dental embryonic remnants. An extremely rare extragnathic localization of AC, ectopic ameloblastic carcinoma (EAC), arises from tissues with no direct connection to the jaws. Only three cases of AC with extragnathic localization have been described in the literature (1-3). This report presents a patient with an EAC arising from the skull base. Taking into consideration new scientific discoveries on the molecular pathogenesis of ameloblastoma (4), we are the first to have performed BRAF mutation analysis in an EAC patient. Due to the tumor’s extremely rare localization and diagnostic challenges, our case study may be a valuable addition to the literature.

## CASE REPORT

**Figure 1:**
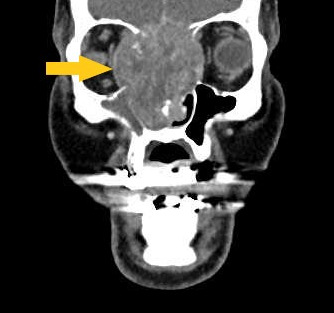
Preoperative multi-slice computed tomography (CT). The arrow points to the tumor.

A 64-year-old man was referred to our Department for a second opinion after having been diagnosed with a non-intestinal-type adenocarcinoma in the nasal cavity. The patient had no history of chronic or hereditary diseases. The symptoms included difficulty breathing through the nose and tearing of the right eye alongside with double vision. He had a visible protrusion of the right eye, bulbar dystopia to the right, and diplopia in all directions. The inspection of the nose revealed a large mass in the nasal cavity. Multi-slice computerized tomography (CT) and magnetic resonance imaging (MRI) showed a mass in the nasal cavity and ethmoid bone, with propagation to both orbital cavities and intracranial extension through the lamina cribrosa involving basal parts of both frontal lobes of the brain. The mass was about 7 cm in its greatest dimension and it infiltrated the right frontal and sphenoid sinuses, leaving the maxillary sinuses tumor-free (Figure 1). Radiological imaging revealed no evidence of metastasis. The patient had never been exposed to radiation (therapeutic or otherwise).

**Figure 2:**
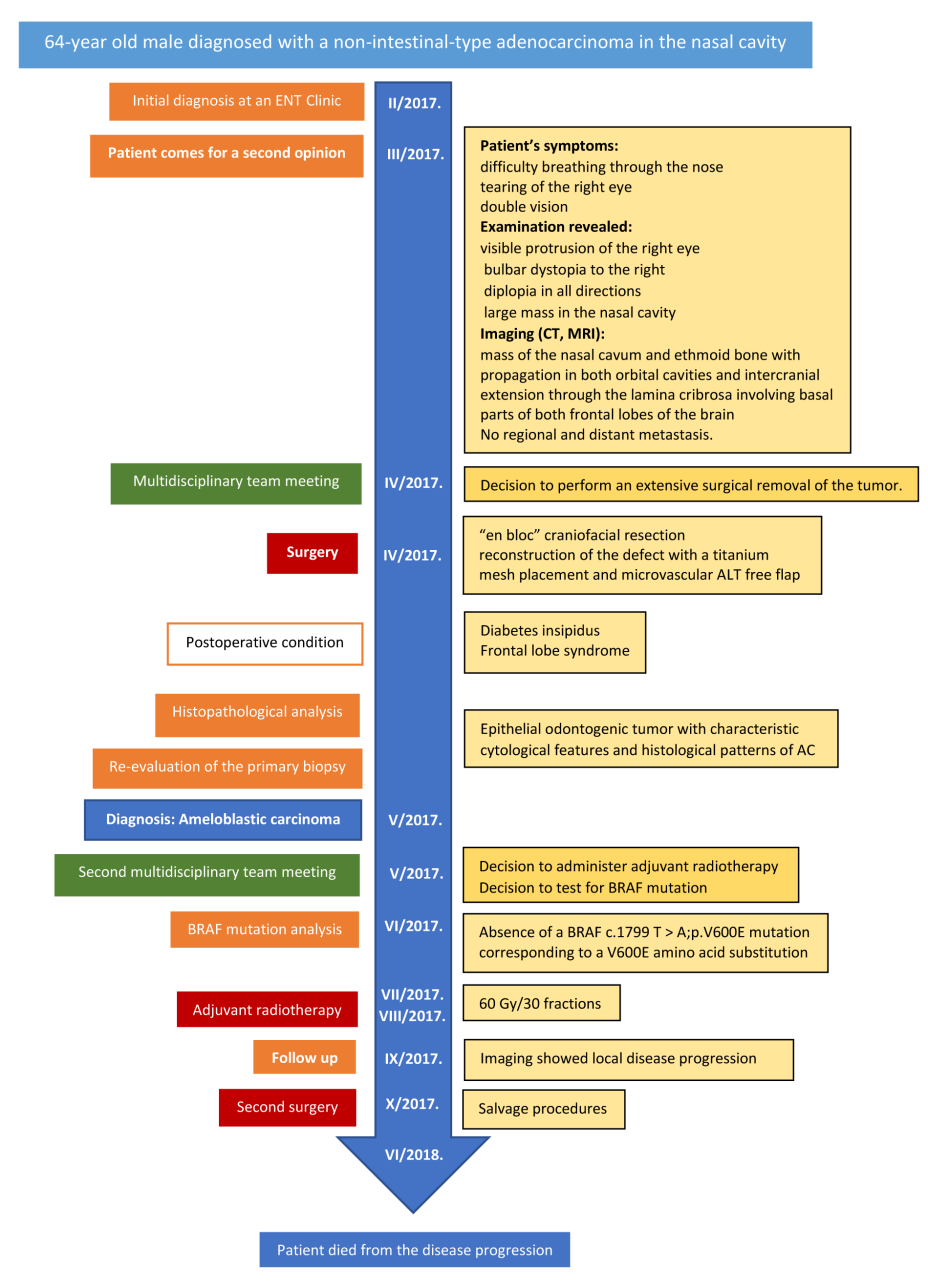
The timeline of diagnostic and clinical procedures. ENT – ear, nose and throat; CT – computerized tomography; MRI – magnetic resonance imaging; ALT – anterolateral thigh, AC – ameloblastic carcinoma.

A craniofacial resection of the tumor consisted of a medial right maxillectomy with the exenteration of the right orbit, followed by a resection of the frontal glabellar bone, nasal bones, and roof and medial wall of the right orbit. Postoperatively, the patient developed diabetes insipidus and frontal lobe syndrome (Figure 2).

**Figure 3:**
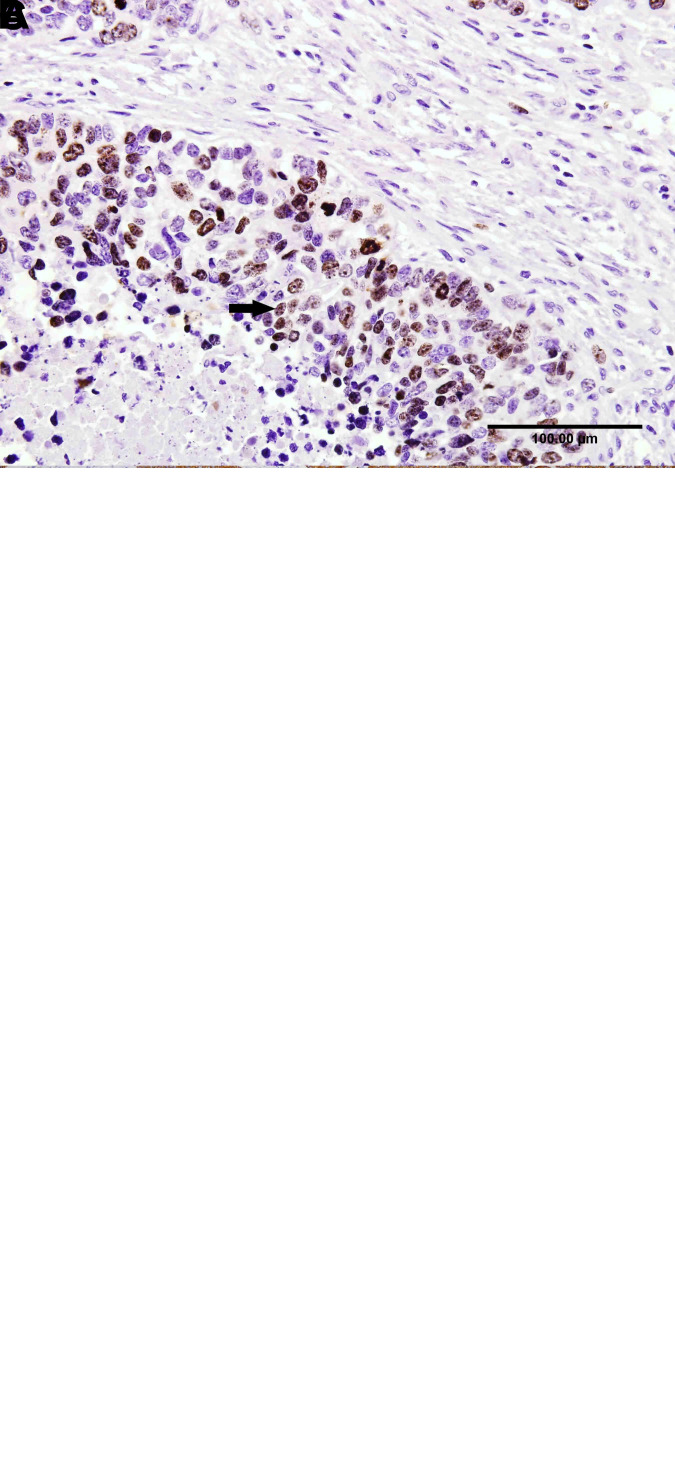
Histopathologic features of ameloblastic carcinoma. (**A**) Black arrow points to the peripheral basal layer of palisading cells and orange arrow points to the stellate reticulum-like central epithelium with numerous mitotic figures and cellular atypia (hematoxylin and eosin stain – 400 × magnification). Scale bar: 100 μm. (**B**) High-intensity immunostaining for CK18 (100 × magnification). Scale bar: 200 μm. (**C**) High Ki-67 proliferation rate (400 × magnification). The arrow points to the Ki-67 positive cell with mitotic activity. Scale bar: 100 μm.

Histopathological and immunohistochemical analysis showed an epithelial odontogenic tumor with characteristic cytological features, histological patterns of AC with perineural and vascular invasion, high CK18 expression, and high proliferation index Ki-67 (Figure 3A, 3B, and 3C). Upon reevaluation, the primary biopsy specimen showed the same histopathological pattern.

BRAF mutational analysis with an allele-specific polymerase chain reaction test revealed the absence of a BRAF c.1799 *t* > A;p.V600E mutation (5).

After adjuvant radiotherapy (TD = 60 Gy/30 fractions), no residual tumor was evident. Unfortunately, the follow-up imaging five months later showed local disease progression. Salvage procedure reduced extracranial skin tumor masses, but the patient died 14 months after the first surgery.

## DISCUSSION

Ameloblastic carcinoma amounts to less than 1% of all tumors of the jaw. AC is more common in men, particularly in their fourth and seventh decade of life (male to female ratio, 2-4:1). Two thirds of ACs are primarily located in the mandible, especially in its posterior region, and about a third are located in the maxilla. The local recurrence rate is 28%, and metastasis rate is 34.6% (6).

A literature research identified three cases of EAC. The rarity of AC itself makes EAC an extremely rare occurrence, which is only now described as an isolated phenomenon. The four cases described in this review make up 2% of 200 reported AC cases (Table 1). Although the etiology of EAC is unclear, several potential origins have been proposed: 1) odontogenic epithelium captured in the sinonasal tract during palate formation, 2) sinonasal cells acquiring the potential of odontogenesis during development, 3) ectopic teeth as a potential origin of odontogenic tumors, 4) malignant transformation of adamantinomatous craniopharyngioma (3,7). However, one of the case reports (2) described EAC development in the neck, which is not consistent with these mechanisms but may point to the tumor origin in the ectopic odontogenic epithelium in the neck. Tumor rarity and a variety of histopathological patterns make the AC diagnosis very challenging, especially for the pathologists inexperienced in odontogenic pathology. This challenge is even greater with EAC, as can be seen from the fact that two of the three patients in other reports, and our patient, were initially misdiagnosed, and one report did not provide information on the primary biopsy. The main differential diagnosis of AC is ameloblastoma since both tumors have the same histological pattern – a stellate reticulum-like central epithelium and peripheral basal layer of palisading cells with reverse nuclear polarity. The difference is in the presence of cellular atypia and high mitotic activity observed in AC. A useful tool in the diagnosis of AC is immunohistochemical analysis, as 70% of the cases show moderate to strong CK18 expression, while 80% of ameloblastomas have negative reactivity for this cytokeratin. AC also has noticeably higher Ki-67 proliferation rate (13.4%-21.4%) than ameloblastoma (6.4%), indicating its aggressive behavior (8). The occasional presence of spindle cells makes AC difficult to be distinguished from carcinosarcomas and sarcomas. Another similar tumor type, adamantinomatous craniopharyngioma, as opposed to AC, shows focal keratinization and much lower mitosis and proliferative index, as well as less expressed inverted nuclear polarity. In addition, craniopharyngiomas usually develop in the suprasellar region, very rarely intranasally. In our case, the mentioned clinical, pathological, and immunohistochemical characteristics were very prominent (strong expression of CK18, high proliferation rate and characteristic pathohistological appearance of AC, infrasellar localization). Importantly, the specimens were examined by two experienced pathologists specialized in head and neck tumors.

**Table 1:** Clinical characteristics of all patients with ectopic ameloblastic carcinoma (EAC) reported in the literature

Authors /y	Sex	Age (years)	Location of EAC	Symptoms	Initial biopsy	Imaging examination	Surgery	Radiotherapy	Recurrence	BRAF V600E	Follow-up (months)	Outcome
**Ozlugedik et al** **2005**	F	23	anterior skull base	severe headache, swelling on the nasal root	squamous cell carcinoma	CT, MRI	+	+	+	NA	25	no evidence of disease
**Gao et al** **2010**	F	42	intrasellar region	decreased visual acuity, headache	craniopharyngioma	MRI	+	-	+	NA	13	patient died
**Hong et al** **2019**	M	54	upper neck	submandibular node	NA	PET-CT	+	+	-	NA	NA	no evidence of disease

The most commonly used method in AC treatment is radical surgical excision aiming to obtain negative margins, with the proposed margin width ranging from 1 to 3 cm. Giridhar et al (9) designed a possible treatment algorithm for AC, suggesting that all patients with high-risk factors (R1 resection, node positive and recurrent tumor, over 45 years old) should undergo adjuvant radiotherapy at a dose of 60 Gy. Since our patient had positive resection margins and age older than 45, he was administered this treatment after surgery. There is no sufficient clinical data to establish the prognosis of EAC, but available information for AC suggests a modest 5-year overall survival of 69% (9).

The weak response of AC to chemotherapy and radiotherapy requires the use of adjuvant treatment options (9). New scientific discoveries on the molecular pathogenesis of AC (mitogen-activated protein kinase and sonic hedgehog pathways) may play a major role in developing treatment protocols, including personalized therapy. The molecular-target therapy research has focused on BRAF mutations, since 63%-82% of patients with ameloblastomas and 38% of patients with AC have a BRAF V600E mutation. So far, BRAF inhibitors have not been used in the treatment of AC, but in a few cases dabrafenib and trametinib significantly reduced the size of stage-IV ameloblastoma (10). Our patient is the only EAC patient reported to have been tested for BRAF mutation. Due to EAC rarity and the initial misdiagnosis in almost all cases, we believe that our case represents a valuable addition to the literature, especially since BRAF-positive tumors continue to be a potential target for adjuvant treatment.
